# Improving Consistency of Photobiomodulation Therapy: A Novel Flat-Top Beam Hand-Piece versus Standard Gaussian Probes on Mitochondrial Activity

**DOI:** 10.3390/ijms22157788

**Published:** 2021-07-21

**Authors:** Andrea Amaroli, Praveen Arany, Claudio Pasquale, Stefano Benedicenti, Alessandro Bosco, Silvia Ravera

**Affiliations:** 1 Department of Orthopedic Dentistry, Faculty of Dentistry, First Moscow State Medical University (Sechenov University), 119991 Moscow, Russia; 2 Department of Surgical and Diagnostic Sciences, University of Genoa, 16132 Genoa, Italy; clodent@gmail.com (C.P.); stefano.benedicenti@unige.it (S.B.); 3 Departments of Oral Biology, Surgery and Biomedical Engineering, University at Buffalo, Buffalo, NY 14260, USA; prarany@buffalo.edu; 4 Bosco Ottica S.r.l., Castel Rozzone, 24040 Bergamo, Italy; alessandro.bosco@boscoottica.it; 5 Department of Experimental Medicine, University of Genoa, 16132 Genoa, Italy; silvia.ravera@unige.it

**Keywords:** phototherapy, light therapy, low-level laser therapy, ATP, mitochondria respiratory chain, light-emitting diode, near-infrared light, energetic metabolism

## Abstract

The tremendous therapeutic potential of photobiomodulation therapy in different branches of medicine has been described in the literature. One of the molecular mechanisms for this treatment implicates the mitochondrial enzyme, cytochrome C oxidase. However, the efficacy and consistency of clinical outcomes with photobiomodulation treatments has been fiercely debated. This work was motivated by this need to improve photobiomodulation devices and delivery approaches. We designed a novel hand-piece with a flat-top beam profile of irradiation. We compared the beam profile versus a standard hand-piece and a fibre probe. We utilized isolated mitochondria and performed treatments at various spots within the beam, namely, the centre, left and right edge. We examined mitochondrial activity by assessing ATP synthesis with the luciferin/luciferase chemiluminescent method as a primary endpoint, while mitochondrial damage was assessed as the secondary endpoint. We observed a uniform distribution of the power density with the flat-top prototype compared to a wide Gaussian beam profile with the standard fibre and standard hand-piece. We noted increased production of ATP in the centre of all three beams with respect to the non-treated controls (*p* < 0.05). Both the fibre and standard hand-piece demonstrated less increase in ATP synthesis at the edges than the centre (*p* < 0.05). In contrast, ATP synthesis was increased homogenously in the flat-top handpiece, both in the centre and the edges of the beam. Fibre, standard hand-piece and the flat-top hand-piece prototype have discrete beam distribution characteristics. This significantly affected the mitochondrial activity with respect to their position within the treated areas. Flat-top hand-piece enhances the uniformity of photobiomodulation treatments and can improve the rigour and reproducibility of PBM clinical outcomes.

## 1. Introduction

The ability of visible and near-infrared light (NIR) to influence body healing has been described by several ancient civilizations [1]. The first scientific description by Prof. Endre Mester about fifty years ago outlined the effects of low dose laser interaction with tissues describing the non-thermal therapeutic benefits of biophotonics energy [2,3,4]. Since then, growing evidence has demonstrated that visible and near-infrared light can modulate metabolism in various life forms, from bacteria and protozoa to animals and humans [5]. While all life forms need energy for survival, unlike plant-cell, the animal cell does not appear to directly employ light as a metabolic source of energy [6]. Nonetheless, there are several specialized cells in the human body capable of photoreception that harness light to enable vision and circadian rhythm [7]. Additionally, several biological molecules are capable of interacting with visible and NIR light wavelengths. These include oxyhaemoglobin, melanin, cytochrome and metalloproteins, sulphur-protein, water and lipids [8].

A major site for light interaction has been noted to involve the mitochondria [8]. These interactions can result via direct interaction of cytochromes belonging to the respiratory chain. Alternatively, they can indirectly impact biophysical properties of water, lipids and voltage-gated ion channels as well as changes in calcium homeostasis and membrane fluidity [8,9,10]. In other words, the photon can transfer its energy to the photoacceptor in the mitochondria that leads to an electronically excited state and production of energy substrates such as ATP [11,12]. Thus, this ability to modulate the cellular metabolism and functions via non-ionizing and non-thermal light treatments is termed photobiomodulation (PBM) therapy; popularly termed low-level light/laser therapy or cold laser treatments.

A growing literature has noted the popularity of photobiomodulation therapy in discrete branches of medicine from performance enhancement, supportive cancer care to neurorehabilitation. There has been significant recent progress in our understanding of PBM mechanisms, extending beyond the initial effects on the mitochondria to cell membrane receptors and ion transporters as well as an extracellular growth factor, TGF-β1 [13]. However, the standardization and the repeatability of the photobiomodulation process is currently under debate [1,9,10,11,13,14,15]. Two major parameters that can influence this have been identified. First, the variation in tissue optical properties such as scattering due to their microstructure, wavelength-dependent absorption of photon-energy, skin colour and thickness affects the transmittance and reflectance of laser light [16,17,18]. These parameters are not assessed directly in vitro lab studies. Therefore, a coherent translation of the results from in vitro to clinical patients is limited. Second, the photobiomodulation parameters do not directly correlate with precise light and cellular photoacceptor interaction. These can drastically vary with even small changes in the photon-energy delivered [11]. 

Hanna et al., [19] recently demonstrated that by moving the hand-piece from contact to many centimetres away from the target, the treatment power varies with the use of a standard gaussian hand-piece with respect to a flat-top beam delivery system. The same authors also reported improved cell growth and differentiation with treatments using the flat-top hand-piece than the standard probe. They attributed this response to the more homogenous power distribution within the treatment area through the flat-top probe. However, this has not yet been experimentally demonstrated. This study was designed to address this issue. We examined the beam profiles of a novel hand-piece with a flat-top beam profile compared to a standard hand-piece and fibre probe. Next, we investigated the effects of the three probes on isolated mitochondria by assessing ATP generation in various areas of the laser beam, namely in the centre and at the edges on either side. Finally, we also examined the effects of various treatment doses with the three probes on potential mitochondrial damage.

## 2. Materials and Methods

### 2.1. Experimental Design and Purpose

The experiments followed standardized methods for mitochondria isolation, PBM treatments and ATP synthesis evaluation as noted in our prior works with a few modifications [11,20,21]. We aimed to compare the effectiveness of laser treatments on the entire treatment spot-size area. The primary endpoint was the impact of the beam power distribution on mitochondrial activity. The secondary endpoint was the induction of mitochondrial damage. An iLux, Real-Time MecOS 2.0, (Mectronic Medicale S.R.L., Grassobbio, Bergamo, Italy) was employed to perform treatments through probes such as fibre, standard hand-piece and the novel flat-top hand-piece prototype.

**Figure 1:**
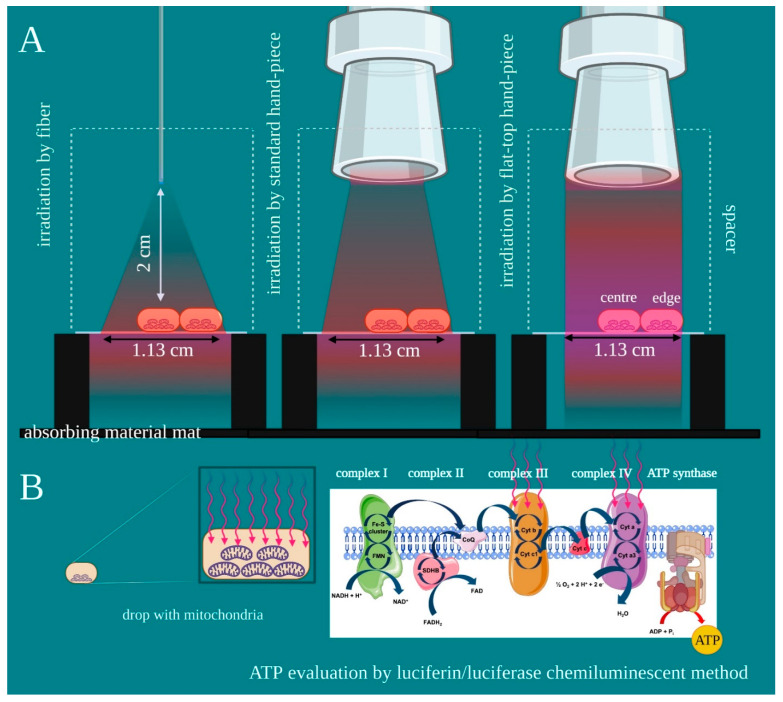
Experimental design. (**A**) A drop of isolated mitochondria enriched-fraction was pipetted on a coverslip, in the centre or the edge of the laser beam circular area, according to the experimental purpose. The drop was then irradiated with an 808-nm laser light (diameter 1.13 cm; spot-size 1 cm^2^) through a fibre, a standard hand-piece, or the flat-top hand-piece. (**B**) Primary and secondary endpoints were evaluated as ATP synthesis through the luciferin/luciferase chemiluminescent method. Image created with BioRender.com.

As the flat-top hand-piece keeps the spot-size area constant from contact to many centimetres [19], this was kept constant at 1 cm^2^ with all probes set up. A 635-nm red light pointer (>0.5 mW) was used to measure probe spot-size areas on a graph, and the correct distance to obtain it was evaluated with a spacer (2 cm) (Figure 1A). Therefore, a circular area of 1 cm^2^ was outlined on a square microscope coverslip for transmitted light microscopy (2.4 cm on each side). The coverslip had a thickness of 0.13 mm and optical properties in accordance with ISO 8255 and ISO 8255-1. The two sides of the coverslip were suspended at 3 cm from an absorbing material mat positioned at the bottom.

A 50 μL drop, approximatively 1/3rd of laser beam, of isolated mitochondria enriched-fraction, was pipetted in the centre or on edge (left and right) (Figure 1). The circular area was then illuminated with an 808-nm laser light (spot size 1 cm^2^) through a fibre, a standard hand-piece, or the flat-top hand-piece prototype. A power meter Pronto-250 (Gentec electro-optics, Inc. Canada) was employed to monitor the power at the target and 2 cm from away. Then, the laser with power set at 1 W, which allowed for the generation of the power density of 1 W/cm^2^ and energy and fluence of 60 J and 60 J/cm^2^, respectively. The treatments were performed in continuous wave mode for 60 s. For the controls, treatments were performed with the same experimental set-up, but with the device switched off (0 W, 60 s). The laser parameter was chosen in accordance with our prior work on isolated mitochondria [21]. Due to possible undesirable thermal effects, adverse events were avoided by monitoring the irradiation with a thermal camera FLIR ONE Pro-iOS (FLIR Systems, Inc. designs, Portland, OR, USA, dynamic range: −20 °C/+400 °C; resolution 0.1 °C) during treatments. Our previous study showed the power of 1 W treatments with similar parameters stimulates the mitochondria complex III and IV [21]. This study examined ATP production with the luciferin/luciferase chemiluminescent method (Figure 1B). The reliability of the experimental set-up was evaluated with the dosimetry for the isolated mitochondria model, described by our team in previous work [22]. To avoid operator bias, the treatments and the data analyses were performed by different operators in a blinded manner.

### 2.2. Design of the Flat-Top Hand-Piece 

**Figure 2:**
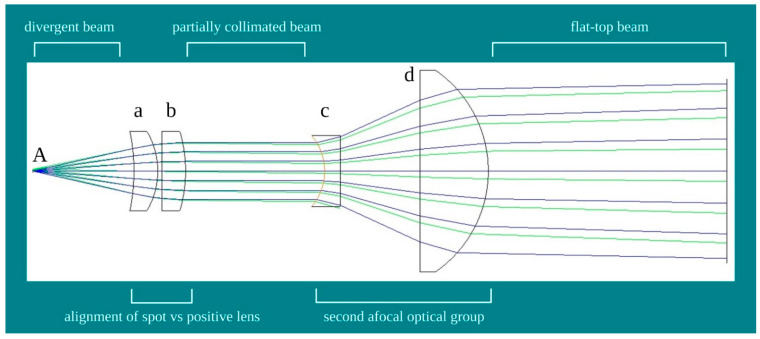
Schematic design of the flat-top hand-piece. (**A**) = fibre’s spot delivering laser light; (**a**,**b**) = collimating lens; (**c**,**d**) = couple of lens to generate an afocal optical group.

The prototype of the hand-piece with flat-top beam profile relies on the probe with an international industrial patent (n.00001425863). Basically, fibre and hand-pieces employed in laser treatments have a non-uniform distorted beam profile resulting in their divergent power density distribution [21,23]. Improvements may be obtained through beam collimation with an alignment between the fibre’s spot delivering light (Figure 2A) and the focal point of a positive lens (Figure 2a,b). However, this approach does not ensure true collimation due to two issues. The first issue is that a large optical fibre size has a longer focal length of the lens resulting in a more diffractive effect. Hence, the beam is not truly collimated, but tends to diverge (partially collimated). The second issue is the fibres routinely used in devices for phototherapy. The optical fibre may transmit light radiation through a series of continuous internal reflections across its length. This feature may support the homogeneous distribution of the power density on the spot size enabling reasonable non-distortive magnification to expand the spot size. However, these critical reflections require fibres parameters incompatible with current clinical devices.

To improve the distribution of the photons on the spot-size area and meet the features of the iLux, real-time MecOS 2.0, a novel integrated optical system was introduced (Figure 2c,d). This system does not use filters or artifices to modify photon intensity and density, and generates a standard Gaussian beam energy profile. A simplified scheme of the novel hand-piece with a flat-top beam profile, project PRG004.20.02 (Bosco Ottica srl, Castel Rozzone, Bergamo, Italy), is shown in Figure 2. First, the alignment of the fibre’s spot delivering light (Figure 2A) with the focal point of the positive lens (Figure 2a,b) generates partial collimation. Then, a second afocal optical group was introduced (Figure 2c,d), which reduces the residual divergence and improves the collimation of the beam and, consequently, the flatness of the wavefront.

### 2.3. Characterization of the Probes Beam Profile

The power density distribution of the beam was characterized with a camera Spiricon SP928 equipped with the software BeamGage Professional (Ophir Spiricon Europe GmbH, Darmstadt, Germany). The measuring tool shows a palette of colours; the power density was measured in a point reflected as purple = 0.1 W/cm^2^; fuchsia = 0.3 W/cm^2^; blue = 0.6 W/cm^2^; light blue = 0.9 W/cm^2^; green = 1 W/cm^2^; yellow = 1.6 W/cm^2^; orange = 2.0 W/cm^2^; Red = 2.2 W/cm^2^.

### 2.4. Mitochondria Enriched-Fraction Isolation

Bovine liver from two males and females was acquired at the slaughterhouse, Ceva, Torino, Italy. These specimens were less than 1 year old and were bred for human consumption following the directives of the Italian Ministry of Agricultural, Food and Forestry Policies. As the animals were not bred or sacrificed at the University of Genoa, ethical committee approval was deemed unnecessary. Specimens were collected and immediately processed after slaughter, following all safety rules. To isolate mitochondria enriched fraction, the bovine livers were washed in PBS and homogenized in a buffer solution containing: 0.25 M sucrose, 0.15 M KCl, 10 mM Tris-HCl pH 7.4 and 1 mM EDTA. The homogenate was then centrifuged at 800× *g* for 10 min. The supernatant sample was filtered and centrifuged at 12,000× *g* for 15 min. The pellet was resuspended in another buffer containing: 0.25 M sucrose, 75 mM mannitol, 10 mM Tris-HCl pH 7.4, 1 mM EDTA. Lastly, the supernatant was centrifuged at 12,000× *g* for 15 min and the mitochondrial pellet was resuspended in the same buffer [11,20,21,24].

### 2.5. Evaluation of Mitochondrial ATP Synthesis

To evaluate the ATP production through the ATP synthase (Fo-F1 ATP synthase), mitochondria enriched-fraction treated with or without the 808-nm laser were diluted in a solution containing: 100 mM Tris-HCl pH 7.4, 100 mM KCl, 1 mM EGTA, 2.5 mM EDTA, 5 mM MgCl_2_, 0.2 mM di(adenosine-5′) penta-phosphate, 0.6 mM ouabain, ampicillin (25 µg/mL), 5 mM KH2PO4 and 5 mM pyruvate + 2.5 mM malate, used as respiratory substrates. The ATP synthesis started after the addition of 0.1 mM ADP and was monitored for 2 min, in a luminometer (Glomax 20/20, Promega, 20126 Milan, Italy) by the luciferin/luciferase chemiluminescent method [11,20,21,23]. An ATP standard solution between 10^−9^ and 10^−7^ M was used for calibration.

### 2.6. Statistical Analysis 

Statistical analyses were performed with GraphPad Prism software version 7 (GraphPad Software, La Jolla, CA, USA). All parameters were tested by one-way ANOVA followed by the Bonferroni test. Data are expressed as mean ± standard deviation (SD) from 3 to 5 independent determinations performed in duplicate. In the figures, SD is shown as error bars. An error probability with *p* < 0.05 was selected as significant.

## 3. Results

### 3.1. Characterization of the Probes Beam Profile and Irradiation

**Figure 3:**
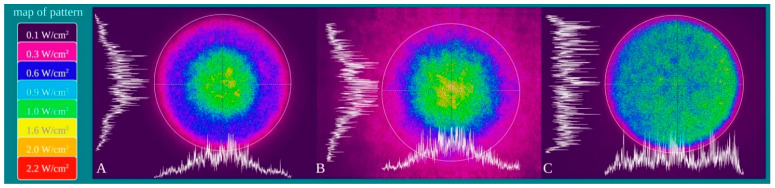
Characterization of the fibre (**A**), standard hand-piece (**B**) and the novel flat-top hand-piece prototype; (**C**) beam profile through a camera Spiricon SP928 equipped with the software BeamGage Professional. Setting the instrument for an irradiation with 1 W the main colours displayed are: purple = 0.1 W/cm^2^; fuchsia = 0.3 W/cm^2^; blue = 0.6 W/cm^2^; light blue = 0.9 W/cm^2^; green = 1 W/cm^2^; yellow = 1.6 W/cm^2^; orange = 2.0 W/cm^2^; red = 2.2 W/cm^2^.

**Figure 4:**
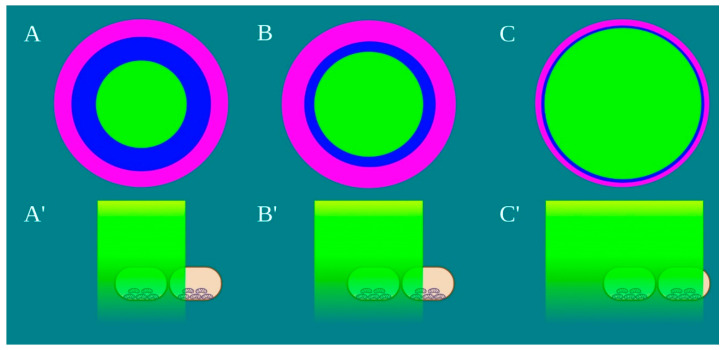
Schematic representation of the laser beam profile (**A**–**C**) and the drop’s area irradiated by the power density of 1 W/cm^2^, green colour, (**A’**–**C’**) Image created with BioRender.com.

Power density is differentially distributed when 1 W of power (1 W/cm^2^ power density) is irradiated through a fibre (A), a standard hand-piece (B) and the flat-top hand-piece (C) within the 1 cm^2^ treatment spot area (Figure 3). As indicated in the material and methods section and on the figure, different colours indicate differences in power density distribution. The green colour (1 W/cm^2^) is distributed on only the 27–30% and 39–41% of the irradiated area when a fibre or a standard hand-piece was employed (Figure 3 and Figure 4). The remaining areas demonstrated power density in the range of 0.6–0.1 W/cm^2^. Conversely, a wider area of ~90% of green colour was described during irradiation through the flat-top hand-piece. Additionally, in the centre of both the spot sizes generated through fibre and standard hand-piece irradiations, higher energy distribution was pointed out by red, orange and yellow colours. Therefore, a distribution of the power density such as a wide-Gaussian beam profile can be considered for fibre and standard hand-piece, compared with a more uniform profile observed with the flat-top prototype.

According to Hanna et al. [19] and also in our experimental set-up, the irradiation with the flat-top hand-piece keeps power constant in contact mode and at 2 cm from the target (1.03 ± 0.02 W vs. 1.01 ± 0.03 W; *p* > 0.05). Irradiation with fibre or the standard hand-piece experienced a statistically significant decrease of 0.23 and 0.22 W, *p* < 0.05. A statistically significant increment of the drop temperature was observed after irradiation (~2.1 °C), but the thermal increase was similar with the three probes (*p* > 0.05).

### 3.2. Evaluation of Mitochondrial ATP Synthesis

**Figure 5:**
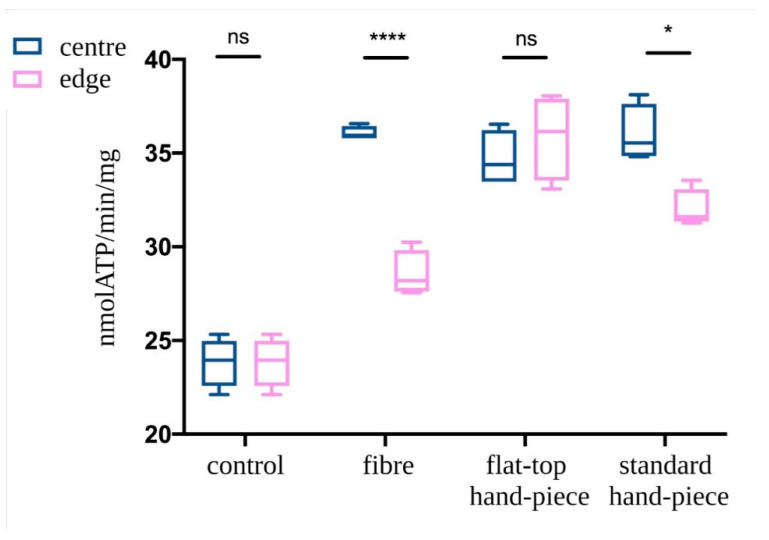
Effect of the photobiomodulation on mitochondrial ATP synthase activity. The isolated mitochondria were placed at the centre or edges of the laser treatment spot (1 cm^2^) and the treatments were performed using a fibre, flat-top hand-piece or a standard hand-piece. All samples were treated with PBM 808 nm laser in continuous wave mode with 1 W, 1 W/cm^2^, 60 J and 60 J/cm^2^ for 60 s. Untreated control samples were placed in a similar set-up with laser at 0 W for 60 s. Data are expressed as mean ± SD. A significant difference between the ATP production of the mitochondria in the centre vs. the edge is indicated by the symbol * and ****, respectively *p* < 0.05 and 0.0001, ns indicates a no-significant difference.

ATP synthesis in isolated mitochondria was assessed following PBM treatments with various probes. The mitochondria in the centre of the laser beam with all three probes demonstrated increased ATP production compared to the non-treated control (*p* < 0.05) (Figure 5). However, PBM treatments performed with fibre or standard hand-piece on the mitochondria placed at the edges of the beam had a significantly less increase in ATP synthesis compared to the centre of the beam (*p* < 0.05). This difference was more evident in the fibre group compared to the standard hand-piece (*p* < 0.05). In contrast, the flat-top hand-piece group demonstrated increased ATP synthesis in both the centre and edges uniformly throughout the beam area.

## 4. Discussion

Photobiomodulation involves changes in cellular metabolism through the transfer of energy from photons to its photoacceptors [8]. The pivotal role of mitochondria in the translation of biophotonic energy to biochemical changes has been previously demonstrated [11,20,21,25,26,27]. However, the rigour of the photobiomodulation clinical outcomes has been questioned. The device technology used for photobiomodulation delivery plays a critical role in improving treatment standardization [24]. Indeed, our data point out that power density delivery is affected by the probes used for these treatments. In this study, we noted that despite both the fibre and standard hand-piece delivering the correct power density, only a surface area less than 50% of the laser spot size was effectively illuminated. Further, the distribution of the different power densities on the treatment spot size was more non-uniform for the fibre compared to a standard hand-piece. Strikingly, flat-top prototype delivered the most consistent power distribution over 90% of the treatment area. Furthermore, the flat-top hand-piece was able to maintain constant power from contact to a couple of centimetres away allowing for improved clinical consistency during PBM treatment delivery as previously reported by Hanna et al. [19]. This evidence supports the notion that the effectiveness of photobiomodulation therapy could be significantly influenced by both the probes employed and the delivery technique of the operator. The significant improvement in mitochondrial activity with the flat-top hand-piece prototype demonstrated a homogenous treatment spot size in the centre and on either sides. This contrasted with the significant differences in the fibre and standard hand-piece groups indicating that the biological responses were most uniformly achieved with the current flat-top prototype. Improvements in the laser device, fibre and collimating procedure with the lens and probe design could further significantly improve the rigour and consistency of PBM clinical treatments.

As noted in our previous work, PBM responses involve complex events resulting from the absorption and scattering of the photons and the generation of an electromagnetic field [22]. This work was based on three-dimensional modelling of photon waves interacting within the mitochondrial droplet generated between the laser to the coverslip. The differences in the beam power density distribution during treatment with the three probes may impact these interactions affecting the overall photobiomodulation responses. Additionally, recent work from our group observed a limited dose range of a 980 nm diode laser, which affects the complexes III and IV as well as ATP production and oxygen consumption of mitochondria [11]. Slight variations (0.1 W) in the treatment power appeared to drastically modulate the photobiomodulation outcomes. Basically, 0.8–1.1 W kept mitochondria coupled and induced increments of ATP production by increments of complex III and IV activities. In contrast, 0.1–0.2 W uncoupled the mitochondria and had an inhibitory effect of ATP synthesis and increment of oxidative stress, while 0.3–0.7 and 1.2–1.4 W did not appear to affect these responses. These data suggest a major limitation in the reproducibility of photobiomodulation responses lie in a non-homogeneous distribution of the laser energy. Variations in an area of only 1 cm^2^ appeared to induce drastically different photobiomodulatory responses (positive, null or negative) in these in vitro studies. Therefore, we would expect significant differences in groups of neighbouring cells and overall tissues when this therapy is used in clinical in vivo scenarios.

This work has some strengths and limitations. A single spot size of 1.13 cm in diameter was used allowing for a limited drop volume of isolated mitochondria sufficient to assess ATP. Further reduction of the drop diameter or an increment of the laser spot-size as well as reducing treatment power could highlight further, perhaps more dramatic, differences. However, the use of three-dimensional dose modelling [22], the temperature monitoring during treatments [11], the standardised ATP synthesis evaluation of treated mitochondria [11,20,21] and the careful assessment of the treatment power at the target surface with a power meter [19], enabled comparisons of the three discrete probe designs and were clear strengths.

## 5. Conclusions

To summarize, our data demonstrated that the fibre, standard hand-piece and the flat-top hand-piece prototype have different beam energy distribution features. These differences significantly affected our primary endpoint, which was mitochondria activity with respect to their position in the treatment spot size. Our second endpoint showed that even at a power of 1 W, there was no damage to mitochondrial function. These results provide evidence that flat-top hand-piece allows improved photobiomodulation treatment reproducibility, especially in clinical scenarios where the distance from the target surface may vary during treatments and the wide affected area needs uniform irradiation to better experience the photobiomodulation effects.

## Data Availability

Data available on request from the authors.
